# SOCIOECONOMIC FACTORS AND LATER LIFE OUTCOMES IN UNEXPECTED DEMENTIA TRAJECTORIES

**DOI:** 10.1093/geroni/igad104.2658

**Published:** 2023-12-21

**Authors:** Yulin Yang, Elizabeth Luth

**Affiliations:** University of California, San Francisco, San Francsico, California, United States; Rutgers University, New Brunswick, New Jersey, United States

## Abstract

Dementia’s uncertain trajectory may complicate its relationship with social determinants of health—race, ethnicity, socioeconomic status—and put individuals who experience unexpected dementia trajectories (e.g., probable dementia at one point, no dementia at a future point) at increased risk of poorer outcomes. This study identifies dementia trajectories in longitudinal survey data, their association with socio-economic factors, and their relationship with later life outcomes. We analyzed weighted data for 9740 adults aged 65+ participating in ≥3 waves of the 2004-2016 Health and Retirement Study. After identifying 5 dementia trajectory patterns, we analyzed the association between sociodemographic factors and dementia trajectories using multinomial logistic regression. We analyzed the relationship between dementia trajectories, socio-economic factors, and three later life outcomes (hospitalization, nursing home admission, and advance care planning) using logistic and multinomial logistic regression. A small percentage (2.2%) of respondents reported an unexpected dementia trajectory. African American and Hispanic respondents and those with lower socioeconomic status had increased risk of unexpected dementia trajectories (vs. no dementia) (e.g., African American RRR: 3.67, 95%CI: 2.30-5.86, p= 0.000; Hispanic RRR: 1.89, 95%CI 1.08-3.30, p=0.26). Older adults with unexpected dementia trajectories had increased risk of nursing home admission (RRR: 4.13, 95%CI: 1.53-11.16, p=0.005), but not hospitalization or advance care planning. Policy and measurement development should proactively address systemic inequities linked to race, ethnicity and socioeconomic status that emerge in and result from dementia trajectory assessments and lived experiences. Interventions should attend to the needs of individuals with unexpected dementia trajectories with respect to institutionalization.

